# Detecting Condylar Lift-Off with a Piezoelectric Smart Knee Replacement

**DOI:** 10.3390/bioengineering13030346

**Published:** 2026-03-17

**Authors:** Brandon D. Hines, Ryan Willing, Steven R. Anton

**Affiliations:** 1 Department of Mechanical & Nuclear Engineering, Tennessee Technological University, Cookeville, TN 38505, USA; bdhines42@tntech.edu; 2 Department of Mechanical and Materials Engineering, University of Western Ontario, London, ON N6A 5B9, Canada; rwilling@uwo.ca

**Keywords:** total knee replacement, smart knee replacement, condylar lift-off, piezoelectric, compartmental force, compartmental center of pressure

## Abstract

Smart knee replacement technology seeks to provide an in vivo method of tracking long-term postoperative joint loads with the goal of identifying clinically relevant phenomena linked to postoperative dissatisfaction in real time. This study evaluated the ability of a piezoelectric compartmental force and compartmental center of pressure sensing total knee replacement to sense condylar lift-off, which is a clinically relevant phenomenon commonly attributed to postoperative dissatisfaction. A commercially available total knee replacement was modified to include six piezoelectric transducers capable of measuring compartmental forces and tibiofemoral centers of pressure on the articular surface of the tibial bearing insert. The smart knee replacement was evaluated with a six-degree-of-freedom joint motion simulator applying a varus lift-off profile. The study demonstrated that the lift-off was evident in both the sensed joint loads and the localized tibiofemoral centers of pressure obtained from the piezoelectric sensing system. The results indicated that the piezoelectric smart knee replacement could be effective for detecting this clinically problematic mechanical issue.

## 1. Introduction

Total knee arthroplasty (TKA) is one of the most common orthopedic surgical procedures performed today, providing relief for patients suffering from debilitating knee pain and loss of function. Approximately one million procedures are performed annually in the United States, with projections showing increases of up to two million annual procedures by 2030 [1,2]. Around 20% of patients report postoperative dissatisfaction as a result of persistent pain or instability [3,4]. In extreme cases, this postoperative dissatisfaction is severe enough to warrant revision arthroplasty. Revision burdens are currently about 10% [5]. It is well documented that the leading causes of aseptic postoperative discomfort are aseptic loosening and instability [6,7,8,9,10]. Condylar lift-off is known to accelerate implant wear and can lead to loosening of the implant [11,12]. The ability to reliably detect such phenomena in a natural setting provides a noninvasive method for early detection of potential implant failure mechanisms.

The primary clinical tool for detecting and tracking condylar lift-off is fluoroscopy, but this technique is accompanied by several limitations. First, fluoroscopy is only available in clinical settings using specialized equipment, which makes it impractical for the majority of TKA patients to utilize and may result in an inaccurate representation of how patients move in their everyday lives. Additionally, fluoroscopy involves the use of ionizing radiation, which carries potential health risks [13]. Finally, multiple fluoroscopic methodologies have been developed for detecting loss of contact between the femoral component and the tibial bearing insert [14,15], but it has been shown that the methods are not always reliable due to assumed thresholds for component lift-off [16].

Smart TKR prostheses seek to provide a method of collecting postoperative in vivo joint information over the life of the implant to provide an alternative to current clinical practices. Information collected by smart TKRs may include joint forces, joint angles, temperature, and pH, among others. In the case of detecting condylar lift-off, the ability to measure compartmental contact forces may enable such detection. Piezoelectric smart TKRs offer a promising sensing solution for in vivo measurement of compartmental tibiofemoral joint forces and centers of pressure (CoPs), while also enabling energy harvesting to provide a self-powered sensing solution [17].

In this study, we experimentally evaluate the ability of a piezoelectric smart TKR sensing system integrated into a commercially available implant system in capturing condylar lift-off. While joint load sensing has been rigorously evaluated by the research community using strain gauges, triboelectric generators, and piezoelectric transducers [17,18,19], this study marks the first evaluation of detecting condylar lift-off with a smart TKR. Condylar lift-off is engendered using a joint motion simulator to apply a varus lift-off profile to the piezoelectric smart TKR prototype. The compartmental forces measured by the piezoelectric sensing system are used to determine if lift-off is indicated during testing. The ability of the smart TKR to recover measurement of compartmental force and tibiofemoral CoPs once contact between the femoral component and the tibial bearing insert is regained is also investigated in this study.

## 2. Materials and Methods

### 2.1. Smart TKR Design

**Figure 1:**
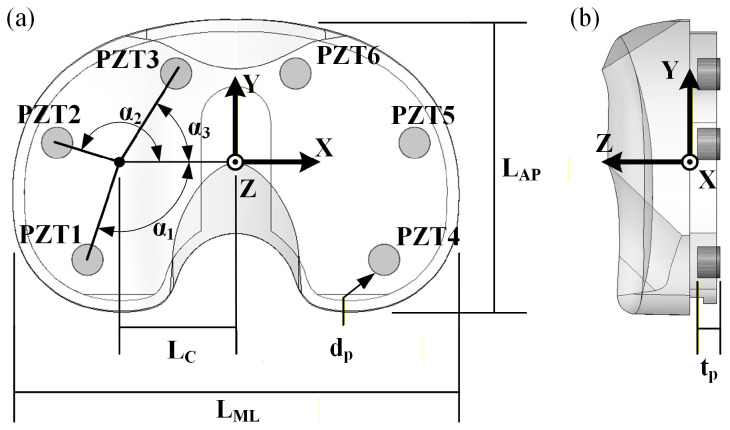
Instrumented tibial bearing insert design parameters shown in (**a**) proximal and (**b**) lateral view.

**Figure 2:**
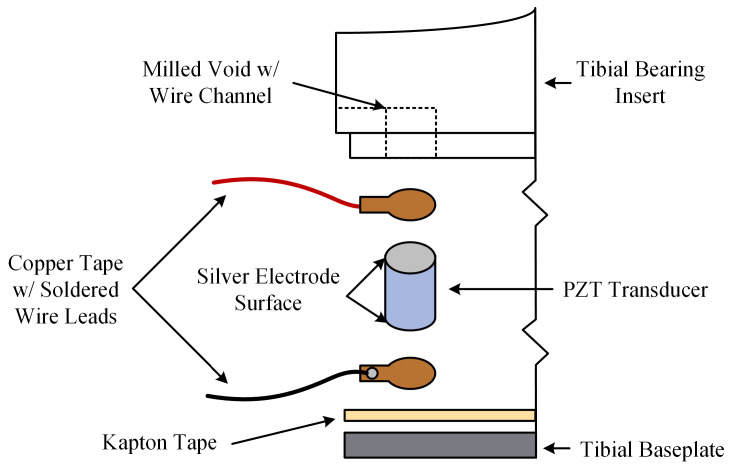
Schematic view of the instrumented smart TKR system.

This study employed a piezoelectric instrumented smart TKR prototype developed by modifying a commercially available TKR. The TKR platform instrumented is the Stryker Triathlon Primary Total Knee system (Stryker Corporation, Kalamazoo, MI, USA). The smart TKR system consists of a size 4 right femoral component and size 3 tibial components. The tibial bearing insert is a 13 mm thick cruciate retaining (CR) X3 highly crosslinked polyethylene with 6 milled voids for embedded lead zirconate titanate (PZT) piezoelectric transducers. The voids were milled such that the embedded piezoelectric transducers lie flush with the original distal surface of the tibial bearing insert. Then, 0.4 mm of material was removed from the entire distal surface, ensuring the load path is channeled through the piezoelectric transducers. The machined tibial bearing insert was fitted with six PZT-5A (APC 850, APC International, Ltd., Mackeyville, PA, USA) piezoelectric transducers, 4.7 mm in diameter by 3.4 mm thick with thin silver electrodes on each parallel surface. The locations of the piezoelectric transducers were selected for optimal force and CoP sensing accuracy, as determined using a parametric computational model and validated through experiments [20]. The design includes three transducers in both the medial and lateral compartments, enabling decoupled measurement of compartmental forces and compartmental centers of pressure (Figure 1 and Table 1). The resulting sensing architecture is analogous to a uniaxial force plate in each compartment, in which distributed sensors are used to reconstruct resultant load and center of pressure from measured forces and moments. Similar principles have been employed in validated commercial intraoperative devices such as VERASENSE [21]. The electroded surfaces of the piezoelectric transducers are in contact with copper tape with soldered wire leads. The tibial baseplate is covered with Kapton tape to ensure each piezoelectric transducer is electrically isolated (Figure 2).

### 2.2. Experimental Setup

**Figure 3:**
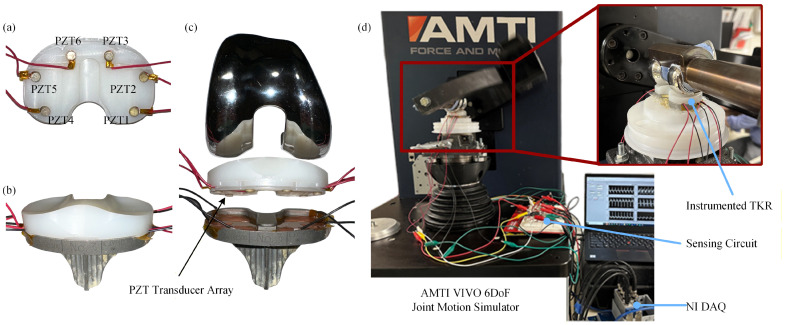
Experimental setup showing (**a**) machined tibial bearing insert with embedded piezoelectric transducers, (**b**) assembled smart TKR prototype, (**c**) exploded view of the components, and (**d**) components cemented into the joint motion simulator.

The tibiofemoral lift-off sensing capability of the smart TKR prototype was evaluated using a six-degree-of-freedom (6-DoF) joint motion simulator (Figure 3; VIVO Joint Simulator, AMTI, Inc., Watertown, MA, USA). The femoral and tibial components were cemented to the femoral and tibial actuators of the joint motion simulator in a neutral alignment (i.e., axis of rotation of femoral component parallel to axis of rotation of femoral actuator, and tibial plateau parallel to horizontal plane, simulating zero posterior tibial slope) using polymethyl methacrylate (PMMA; Fastray, Keystone Industries, Gibbstown, NJ, USA) and gypsum cement (Modern Materials Denstone Golden, Kulzer North America, South Bend, IN, USA), respectively. The components were first brought into contact using the simulator at 0° of flexion. The simulator was then allowed to release its other constraints to settle into a low-energy state (the most distal point on the convex distal femoral condyles resting in the deepest points of the condylar concavities of the tibial bearing) and was zeroed. The joint motion simulator has six degrees of freedom (three translational and three rotational), which can be independently controlled for either force or displacement. Joint loads and kinematics of each DoF were sampled using the joint motion simulator’s internal data acquisition (DAQ) system.

**Figure 4:**
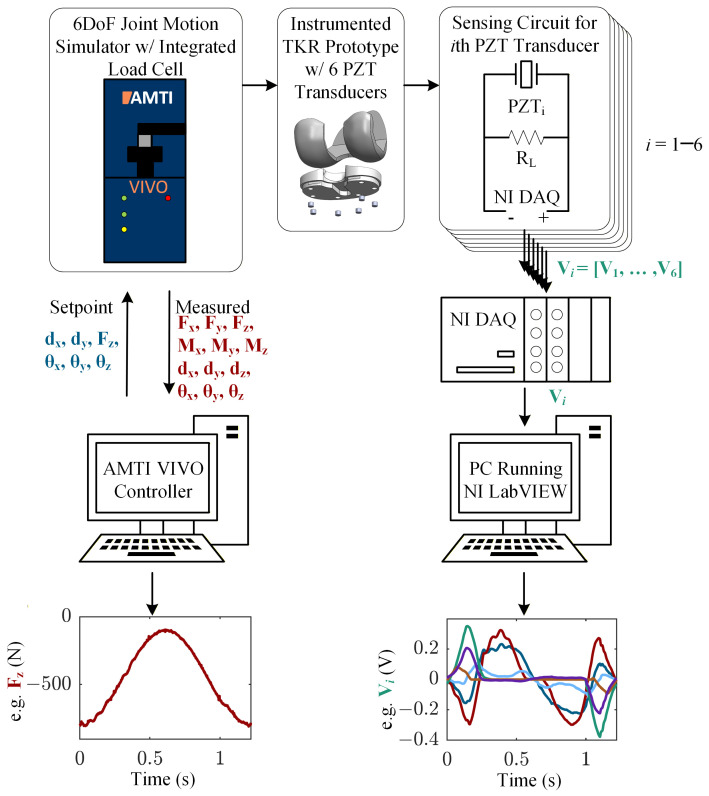
Experimental flowchart showing measurement chain, setpoint variables, and all variables measured during testing.

Each piezoelectric transducer was connected in parallel to a 500 kΩ load resistor ($R_{L}$) (note, the load resistor value was not optimized in this proof-of-concept study; it was selected based on previous testing to ensure that the piezoelectric voltages remained in a measurable range for the DAQ device across a variety of loading scenarios). The voltage response of each piezoelectric transducer was sampled across its respective load resistor with two 16-bit NI 9215 analog input (±10 V) cards inserted into an NI cDAQ-9185 CompactDAQ chassis (National Instruments Corp., Austin, TX, USA). The overall experimental setup consists of the joint motion simulator, the instrumented smart TKR, the measurement circuit, and the National Instruments DAQ system (Figure 4).

**Figure 5:**
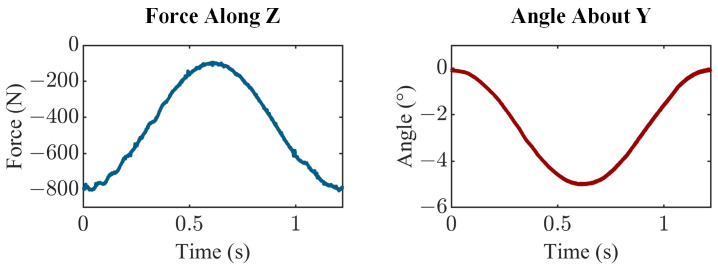
Prescribed degrees of freedom applied to the smart TKR for a representative cycle, as measured by the joint motion simulator.

To achieve varus condylar lift-off (i.e., lift-off of the lateral condyle), the joint motion simulator applied a cyclical varus rotation (0° to 5°) at a rate of 1.22 s per cycle. At 0° of varus (neutral alignment), the joint compressive load was 800 N, but this load decreased to 100 N as varus rotation occurred (Figure 5). This scenario is representative of lateral compartment lift-off when the joint is minimally loaded, with re-engagement when loads are reapplied. The compressive degree of freedom was force controlled, prescribing the sinusoidal compressive load, and the varus-valgus rotational degree of freedom was displacement controlled, prescribing the sinusoidal varus lift-off profile. The medial-lateral and anterior–posterior translations, as well as flexion-extension and internal-external rotations, were constrained to zero. An external excitation was applied to the joint motion simulator to synchronize the two data acquisition systems. The components were lubricated with dish soap before testing. The joint motion simulator applied continuous cycles, and eight consecutive cycles of joint loads and kinematics, as well as the voltage across each piezoelectric transducer, were sampled at a rate of 1000 samples/second.

### 2.3. Modeling and Data Analysis

The voltage generated by a piezoelectric transducer ($V_{i}$) connected to a load resistor ($R_{L}$) under uniform compressive load ($F_{i}$) along the poling direction depends on both the magnitude and frequency of the applied load. When the excitation frequency is far below the mechanical resonance of the transducer, a first-order electromechanical model adequately captures the dynamic behavior of the system. The dynamics of the system can be represented through a circuit modeling approach (for details see Appendix A) or through the piezoelectric constitutive equations [22], each of which results in the electromechanical governing differential equation given by:

(1) $$C_{p}\frac{dV_{i}(t)}{dt}+\frac{V_{i}(t)}{R_{L}}=d_{33}\frac{dF_{i}(t)}{dt},$$

where $d_{33}$ is the piezoelectric strain coefficient, t is time, and $C_{p}$ is the capacitance of the piezoelectric transducer given as:

(2) $$C_{p}=\frac{\varepsilon_{33}^{T}A}{h},$$

where $\varepsilon_{33}^{T}$ is the dielectric permittivity measured under zero stress conditions, A is the cross-sectional area, and h is the thickness. This first-order electromechanical model accounts for the dynamics of the sensing system (again, for frequencies far below the mechanical resonance of the piezoelectric transducer). It is interesting to note that significant voltage attenuation is present at the extremely low frequencies experienced in the lift-off loading profile considered in this study. This is due to the high-pass filtering effect introduced by placing a load resistor in parallel with the piezoelectric transducer. This voltage attenuation is, in fact, required to bring the signal to a range measurable by the data acquisition system. Further details can be found in Appendix B.

For each sampled cycle, the measured voltage response of the $i^{th}$ piezoelectric transducer, $V_{i}(t)$, was converted to piezo-sensed force by numerically solving the first-order electromechanical differential equation (Equation (1)) using MATLAB (The MathWorks, Inc., Natick, MA, USA). Solution of Equation (1) requires an initial condition (i.e., initial force). The six required initial conditions are found by taking the total applied compressive force reported by the joint motion simulator at the beginning of the cycle and distributing it amongst all six piezoelectric transducers according to their respective voltage responses.

The medial and lateral compartmental forces, $F_{M}$ and $F_{L}$, respectively, were determined by summing the individual piezo-sensed forces in each compartment, given as:

(3) $$F_{M}=\sum_{i=1}^{3}F_{i},$$

for the medial compartment, and

(4) $$F_{L}=\sum_{i=4}^{6}F_{i},$$

for the lateral compartment.

The compartmental tibiofemoral CoPs were localized by applying an equilibrium of moments to the individual forces. The x and y coordinates of the medial tibiofemoral CoP, ${{CoP}_{x}}^{M}$ and ${{CoP}_{y}}^{M}$, respectively, are given as:

(5) $${{CoP}_{x}}^{M}=\sum_{i=1}^{3}F_{i}x_{i}/\sum_{i=1}^{3}F_{i},$$

and

(6) $${{CoP}_{y}}^{M}=\sum_{i=1}^{3}F_{i}y_{i}/\sum_{i=1}^{3}F_{i},$$

where $x_{i}$ and $y_{i}$ are the x and y coordinates of the $i^{th}$ piezoelectric transducer. Similarly, the x and y coordinates of the lateral tibiofemoral CoP, ${{CoP}_{x}}^{L}$ and ${{CoP}_{y}}^{L}$, respectively, are given as:

(7) $${{CoP}_{x}}^{L}=\sum_{i=4}^{6}F_{i}x_{i}/\sum_{i=4}^{6}F_{i},$$

and

(8) $${{CoP}_{y}}^{L}=\sum_{i=4}^{6}F_{i}y_{i}/\sum_{i=4}^{6}F_{i}.$$

## 3. Results

**Figure 6:**
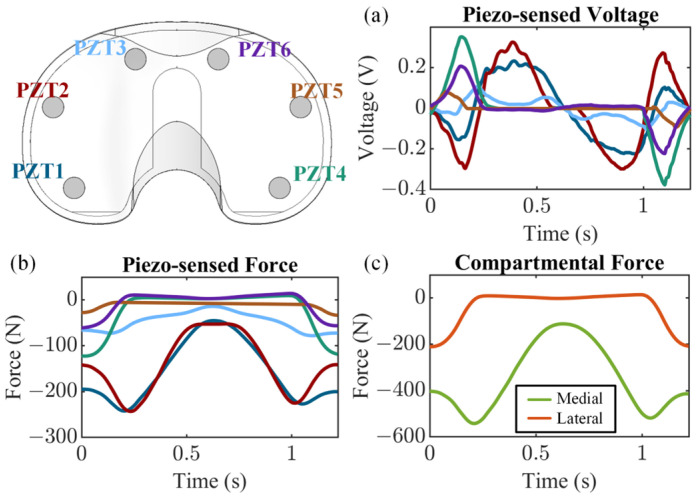
Joint load sensing results from a representative cycle showing (**a**) piezo-sensed voltages and (**b**) computed piezo-sensed forces from each piezoelectric transducer, as well as (**c**) computed compartmental forces (colored lines for part (**a**,**b**) are defined in the upper left image).

The voltage sensed by each piezoelectric transducer from a representative cycle (Figure 6a) was used to compute the force sensed by each piezoelectric transducer (Figure 6b). The transducers embedded in the lateral compartment measured loads of 0 N while lift-off was present (Figure 6b). As the total compressive load increased and contact was regained, the load was then rapidly redistributed to the transducers embedded in the lateral compartment (Figure 6b). Summing the piezo-sensed force in each compartment revealed the rapid transfer of load from the transducers embedded in the lateral compartment (PZT4-PZT6) to the transducers embedded in the medial compartment (PZT1-PZT3) as the femoral compartment lifted off and the total compressive load decreased (Figure 6c).

**Figure 7:**
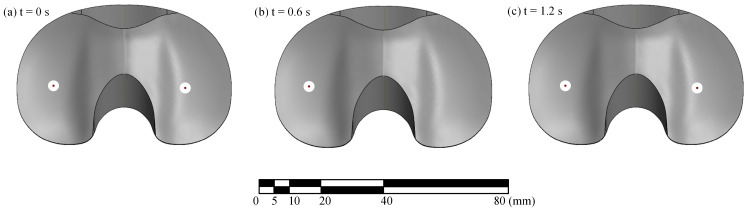
Sensed tibiofemoral CoPs from a representative cycle at (**a**) t = 0 s (in contact), (**b**) t = 0.6 s (lifted off; CoP implausible, therefore not plotted), and (**c**) t = 1.2 s (in contact).

The tibiofemoral CoP localization results (Figure 7) show that as the test began with the lateral compartment in contact, the lateral CoP was at a reasonable location near the center of the condyle (Figure 7a). As the test proceeded and lift-off occurred, the lateral CoP moved to implausible coordinates (i.e., those not located on the surface of the tibial bearing insert; Figure 7b). Once contact was regained, the lateral CoP then moved back to a reasonable coordinate located near the center of the compartment (Figure 7c). It should be noted that during lift-off, the net load in the unloaded compartment approaches zero, resulting in an ill-conditioned CoP calculation due to the division by force as shown in Equations (5)–(8). Although the true compartmental force is effectively zero, the transducers exhibit a small, nonzero response due to electrical noise and slight mechanical coupling from the loaded compartment. When these small residual forces are used in the equilibrium of moments calculation, they can produce disproportionately large CoP deviations that fall outside the articular surface. Accordingly, CoP coordinates during lift-off are non-physical and should be interpreted as undefined (i.e., implausible) until contact is re-established and compartment load returns to appreciable levels.

**Figure 8:**
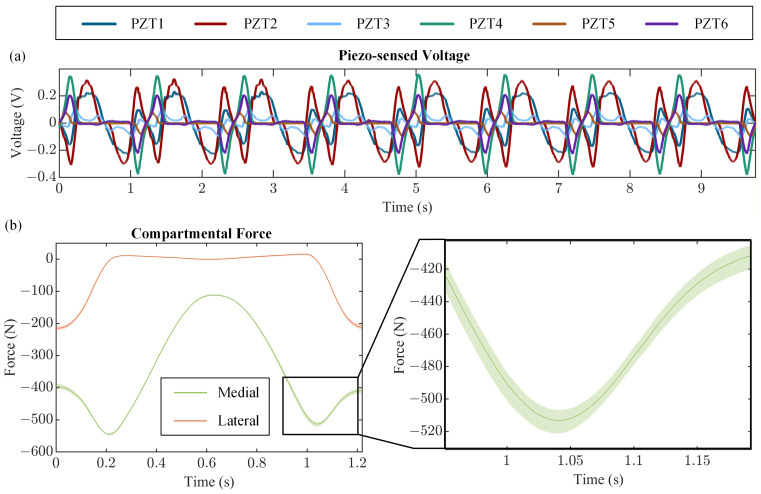
Short-term repeatability results showing (**a**) eight consecutive cycles of sampled voltage and (**b**) average sensed compartmental forces with minimum and maximum envelopes across eight cycles.

Finally, the eight consecutive cycles of sensed voltage (Figure 8a) show short-term repeatability with voltage response remaining consistent across the sampled cycles. The sensed compartmental force demonstrated a maximum spread of ~19 N in the medial compartment and ~14 N in the lateral compartment across the eight sampled cycles (Figure 8b). Overall, these results demonstrate short-term repeatability of compartmental force sensing.

## 4. Discussion

The key findings of this study are that the piezoelectric sensing system was capable of capturing condylar lift-off and recovering force and CoP measurements after joint contact was regained. It was shown that lift-off was evident in both the compartmental joint load measurement as well as the calculated compartmental CoPs. The device was also capable of recovering the measurement once contact was regained. While our device has only been evaluated in vitro and requires further development before implantation into patients (circuit miniaturization, packaging, cadaver testing, life cycle testing, etc.), the ability to detect phenomena such as condylar lift-off in real time provides a reliable metric for assessing implant health without the need for patient compliance, self-reported dissatisfaction, or clinical assessment. Additionally, this metric could allow early identification of issues associated with patient dissatisfaction, thereby allowing physicians to modify recovery plans to prevent dissatisfaction altogether.

The main strength of this study is the real-time capture of condylar lift-off using a piezoelectric smart TKR device capable of measuring compartmental joint loads and tibiofemoral CoPs. This capability offers an alternative to current clinical practices, which are shown to be impractical or unreliable. However, there are limitations of this study that should be considered when interpreting the results. First, while the joint motion simulator used in this study is capable of replicating mechanical aspects of joint function during various ADLs and can even simulate virtual ligaments, the simulator was configured to follow a simple loading profile used to engender condylar lift-off. Thus, the load scenario is over-simplified and not representative of ADLs where lift-off may typically occur. Future research will investigate simulating and detecting lift-off during common ADLs to be more clinically representative. Additionally, expanding to common ADLs will allow the study of transducer performance under higher compressive loads and may offer the opportunity to determine load limits of the transducers.

Furthermore, this study was conducted in vitro; therefore, the data acquisition is external, and the piezoelectric transducers and electrical components are not hermetically sealed. Future work is needed to investigate the miniaturization of the electrical components and packaging of the entire system. An additional limitation of the in vitro nature of this study is that the joint load and tibiofemoral CoP measurements lack the influence of soft tissue effects found in the native knee environment. Future research will also incorporate cadaver studies, which are well documented in the literature, to effectively capture soft tissue effects [23,24,25,26].

Additionally, while short-term repeatability was demonstrated over eight simplified cycles, long-term repeatability and stability were not assessed. Potential effects of signal stability and degradation of the sensors and implant over extended time periods remain uncharacterized. Furthermore, the overall measurement accuracy was not quantified. In particular, the study does not establish an absolute reference (ground truth) for lift-off timing or estimate associated error metrics. Future work should assess the accuracy and long-term repeatability of the smart TKR system.

It is also worth noting that while this study utilized the joint motion simulator to obtain the required initial conditions when solving the governing ODE, future research is required to enable the smart TKR to obtain the initial conditions internally. An alternative path is to investigate data-driven calibration to eliminate reliance on a model (and subsequently the need to obtain initial conditions).

Finally, a single implant model from a single manufacturer was evaluated in this work. Specifically, the implant used features a symmetric design. Asymmetric implant designs are common in total knee replacements, and asymmetry could impact the outcomes. Future studies should investigate a range of implant designs, including asymmetric models.

## 5. Conclusions

This study highlights the effectiveness of a piezoelectric smart TKR system for in vivo measurement of condylar lift-off. The results demonstrated that lift-off was evident in the compartmental joint loads as well as the compartmental CoPs sensed by the smart TKR device. Overall, this work presented a significant step toward the realization of a piezoelectric smart TKR system that can be used as a tool to better understand patient outcomes, allowing for the real-time detection of clinically relevant phenomena related to postoperative dissatisfaction and implant failure.

## Figures and Tables

**Table 1:** Smart TKR relevant dimensions.

Symbol	Value	Parameter
**L_ML_**	67.2 mm	Mediolateral width of the implant
**L_AP_**	44.0 mm	Anteroposterior length of the implant
**L_C_**	18.5 mm	Distance to the angular center of the compartment
**α_1_**	105°	Angle of PZT1
**α_2_**	160°	Angle of PZT2
**α_3_**	55°	Angle of PZT3
**d_p_**	4.7 mm	Diameter of the piezoelectric transducers
**t_p_**	3.4 mm	Thickness of the piezoelectric transducers

**Figure A1:**
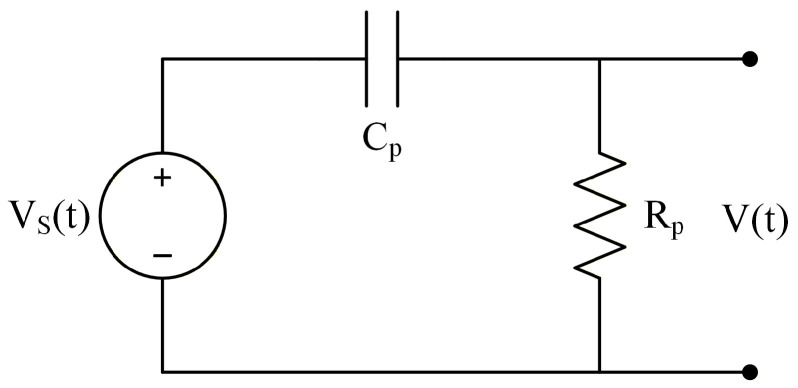
Equivalent circuit of a piezoelectric transducer.

**Figure A2:**
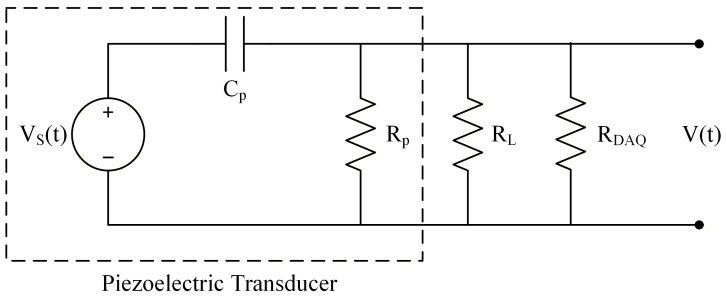
Equivalent circuit of a piezoelectric transducer in parallel with a load resistor and a data acquisition device.

**Figure A3:**
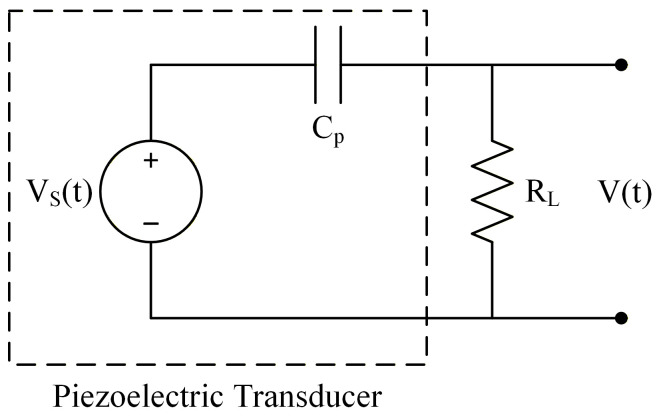
Simplified equivalent circuit of a piezoelectric transducer in parallel with a load resistor.

**Figure A4:**
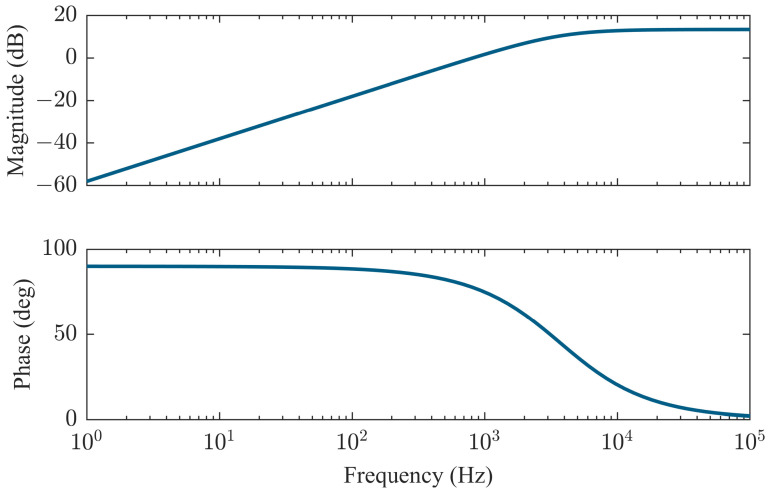
Bode diagram of the piezoelectric system described by Equation (1) (magnitude plotted as $20\cdot {log}_{10}$ of V/N).

## Data Availability

The datasets generated and supporting the findings of this article are obtainable from the corresponding author upon reasonable request.

## Appendix A

A piezoelectric transducer connected to a load resistor and operating far below resonance can be modeled electrically as a voltage source $V_{s}\left(t\right)$ in series with its internal capacitance $C_{p}$ and its internal resistance $R_{p}$ (Figure A1). For sensing, a load resistor $R_{L}$ is connected in parallel to the transducer, and a data acquisition device with internal resistance $R_{DAQ}$ is used to measure the voltage V(t) across the load resistor (Figure A2). The internal resistance of the piezoelectric transducer, as well as the input resistance of the DAQ, is typically much larger than the external load resistor [27], so the behavior is dominated by $R_{L}$, and the circuit simplifies to Figure A3.

Applying Kirchhoff’s voltage law (KVL) to the equivalent circuit gives:

(A1) $$V_{s}\left(t\right)=V_{C}\left(t\right)+V\left(t\right).$$

where $V_{C}(t)$ is the voltage across the transducer capacitance. The current through the capacitor can be represented as:

(A2) $$i\left(t\right)=C_{p}\frac{dV_{C}(t)}{dt},$$

and the current through the load resistor is:

(A3) $$i\left(t\right)=\frac{V(t)}{R_{L}}.$$

Noting that the current through the capacitor and the resistor is equal when connected in series, equating Equations (A2) and (A3) gives:

(A4) $$C_{p}\frac{dV_{C}(t)}{dt}=\frac{V(t)}{R_{L}}.$$

Next, solving Equation (A1) for $V_{C}$ gives:

(A5) $$V_{C}\left(t\right)=V_{s}\left(t\right)-V\left(t\right),$$

and differentiating yields:

(A6) $$\frac{dV_{C}(t)}{dt}=\frac{dV_{s}(t)}{dt}-\frac{dV(t)}{dt}.$$

Now, substituting Equation (A6) into Equation (A4) gives:

(A7) $$C_{p}\left(\frac{dV_{s}\left(t\right)}{dt}-\frac{dV\left(t\right)}{dt}\right)=\frac{V(t)}{R_{L}}.$$

Rearranging gives the governing circuit equation in terms of the output voltage V(t):

(A8) $$C_{p}\frac{dV(t)}{dt}+\frac{V(t)}{R_{L}}=C_{p}\frac{dV_{s}\left(t\right)}{dt}.$$

Next, the source voltage is related to the applied mechanical load. Under a uniform stress approximation for axial loading of a uniform piezoelectric transducer along the poling direction, the piezoelectric voltage is given by:

(A9) $$V_{s}\left(t\right)=g_{33}h\sigma \left(t\right)=g_{33}\frac{h}{A}F(t),$$

where $g_{33}$ is the piezoelectric voltage constant, h is the transducer thickness, A is the electrode area, and $\sigma \left(t\right)=F(t)/A$ is the compressive stress. Using the relationship:

(A10) $$g_{33}=\frac{d_{33}}{\varepsilon_{33}^{T}},$$

Equation (A9) becomes:

(A11) $$V_{s}\left(t\right)=d_{33}\frac{h}{\varepsilon_{33}^{T}A}F\left(t\right).$$

The transducer capacitance can be represented as Equation (2), and substituting Equation (2) into Equation (A11) yields the relation:

(A12) $$V_{s}\left(t\right)=\frac{d_{33}}{C_{p}}F\left(t\right).$$

Taking the derivative gives:

(A13) $$\frac{dV_{s}\left(t\right)}{dt}=\frac{d_{33}}{C_{p}}\frac{dF\left(t\right)}{dt}.$$

Finally, substituting Equation (A13) into Equation (A8) gives:

(A14) $$C_{p}\frac{dV(t)}{dt}+\frac{V(t)}{R_{L}}=d_{33}\frac{dF(t)}{dt},$$

which matches the governing differential equation given by Equation (1).

## Appendix B

The voltage generated by a piezoelectric transducer connected to a load resistor under uniform compressive load depends on both the magnitude and frequency of the applied load. When the excitation frequency is far below the mechanical resonance of the transducer, a first-order electromechanical model adequately captures the dynamic behavior of the system. Analyzing the electromechanical model of the piezoelectric sensing system given in Equation (1), along with the equivalent circuit of the system given in Figure A3, it is clear that the piezoelectric sensing system (i.e., piezoelectric transducer in parallel with a load resistor) exhibits first-order high-pass filter behavior. To see this clearly, the time constant and corner frequency of the system are computed, and the Bode diagram of the system is plotted, as follows.

The piezoelectric system investigated in this study consists of a piezoelectric disk with a diameter of 4.7 mm and a thickness of 3.4 mm, made from APC 850, which has a relative permittivity of $\varepsilon_{r}=1900$, connected to a load resistance of $R_{L}=500$ kΩ. The capacitance of the transducer can be computed from the transducer dimensions and material properties, per Equation (2). First, the permittivity of the transducer is computed as:

(A15) $${\varepsilon_{33}^{T}=\varepsilon }_{r}\varepsilon_{0}=1900\times 8.854e^{-12}\;F/m\approx 1.68e^{-8}\;F/m,$$

where $\varepsilon_{0}$ is the permittivity of free space. Next, the capacitance is computed as:

(A16) Cp=1.68e−8 F/mπ4.7 mm243.4 mm≈86pF.

With the capacitance computed, the time constant of the piezoelectric system (governed by Equation (1) is computed as:

(A17) $${\tau =C}_{p}R_{L}\approx 43\;\mu s.$$

The corresponding corner frequency is:

(A18) $$f_{c}=\frac{1}{2\pi \tau }\approx 3701\mathrm{Hz}.$$

The Bode diagram of the piezoelectric system used in this study is shown in Figure A4. At the extremely low frequencies prescribed in the lift-off profile considered in this study, the high-pass filtering effect of the piezoelectric sensing circuit causes significant voltage attenuation. Again, this voltage attenuation is required to bring the signal to a range measurable by the data acquisition system (as demonstrated in Figure 6), but, as mentioned previously, it should be noted that the load resistor value has not been optimized in this study. A larger load resistance would alter the time constant, corner frequency, and Bode diagram of the sensing system and result in a larger voltage at the data acquisition device.
